# Heterogeneity in White Blood Cells Has Potential to Confound DNA Methylation Measurements

**DOI:** 10.1371/journal.pone.0046705

**Published:** 2012-10-05

**Authors:** Bjorn T. Adalsteinsson, Haukur Gudnason, Thor Aspelund, Tamara B. Harris, Lenore J. Launer, Gudny Eiriksdottir, Albert V. Smith, Vilmundur Gudnason

**Affiliations:** 1 Icelandic Heart Association, Kopavogur, Iceland; 2 Faculty of Medicine, University of Iceland, Reykjavik, Iceland; 3 Laboratory of Epidemiology, Demography, and Biometry, Intramural Research Program, National Institute on Aging, Bethesda, Maryland, United States of America; Geisel School of Medicine at Dartmouth, United States of America

## Abstract

Epigenetic studies are commonly conducted on DNA from tissue samples. However, tissues are ensembles of cells that may each have their own epigenetic profile, and therefore inter-individual cellular heterogeneity may compromise these studies. Here, we explore the potential for such confounding on DNA methylation measurement outcomes when using DNA from whole blood. DNA methylation was measured using pyrosequencing-based methodology in whole blood (n = 50–179) and in two white blood cell fractions (n = 20), isolated using density gradient centrifugation, in four CGIs (CpG Islands) located in genes *HHEX* (10 CpG sites assayed), *KCNJ11* (8 CpGs), *KCNQ1* (4 CpGs) and *PM20D1* (7 CpGs). Cellular heterogeneity (variation in proportional white blood cell counts of neutrophils, lymphocytes, monocytes, eosinophils and basophils, counted by an automated cell counter) explained up to 40% (p<0.0001) of the inter-individual variation in whole blood DNA methylation levels in the *HHEX* CGI, but not a significant proportion of the variation in the other three CGIs tested. DNA methylation levels in the two cell fractions, polymorphonuclear and mononuclear cells, differed significantly in the *HHEX* CGI; specifically the average absolute difference ranged between 3.4–15.7 percentage points per CpG site. In the other three CGIs tested, methylation levels in the two fractions did not differ significantly, and/or the difference was more moderate. In the examined CGIs, methylation levels were highly correlated between cell fractions. In summary, our analysis detects region-specific differential DNA methylation between white blood cell subtypes, which can confound the outcome of whole blood DNA methylation measurements. Finally, by demonstrating the high correlation between methylation levels in cell fractions, our results suggest a possibility to use a proportional number of a single white blood cell type to correct for this confounding effect in analyses.

## Introduction

Tissue and cell specific methylation are well established in human DNA. In 2006 Eckhardt *et al.* presented data from the Human Epigenome Project (HEP, a project that aims to identify, catalog, and interpret DNA methylation profiles of all human genes in all major tissues) that suggest that tissue-specific differentially methylated regions (tDMRs) are very common in the genome [1]. The dataset describes DNA methylation of ∼1.9 million CpG sites on chromosomes 6, 20 and 22 in 12 different tissues. Approximately 22% of the investigated amplicons were tDMRs and their average absolute methylation levels differed by up to 20% between tissues (or up to 15% if only somatic tissues are compared). Recently, Fan and Zhang analyzed DNA methylation in selected (CpG site coverage >30%) CpG islands (CGIs) using the HEP dataset [2]. Similarly, their results indicate that a substantial proportion of CGIs (∼18%) are tDMRs. Three recent independent studies using microarray based methods also identify tDMRs after interrogating CpG sites across the whole genome [3], in CGIs across the genome [4], and in non-CGI regions on chromosome 1 [5].

Relatively few studies have addressed the question whether different white blood cell types have specific DNA methylation levels or patterns. In two papers from 1990 and 1991 Kochanek *et al.* studied the methylation of *TNFα* and *TNFβ* genes in multiple white blood cell types [6], [7]. Their results revealed gross differences in *TNFβ* methylation in lymphocytes versus granulo- and monocytes as well as minor distinctions in the *TNFα* gene between cell types. A comparison of DNA methylation levels in CD4+ and CD8+ lymphocytes was included in the HEP report which showed that these highly developmentally related cell types exhibit on average ∼5% absolute difference in DNA methylation [1]. Finally, Wu *et al.* compared different methods and sources of DNA for measuring global DNA methylation in whole blood [8]. DNA derived from whole blood and two blood fractions (mononuclear cells (MNCs) and polymorphonuclear cells (PMNCs)) was measured using five assays; luminometric methylation assay (LUMA), [^3^H]-methyl acceptance assay and MethyLight assays for long interspersed elements (LINE1), Sat2 and Alu repetitive elements. In four of the five assays, global methylation levels in MNCs and PMNCs were not correlated, suggesting a widespread difference in methylation between the two cell groups.

As peripheral blood cell DNA is relatively easily accessible it has been an essential source for genetic experiments for the past decades. However, whether it is appropriate material for studies on epigenetics has been debated [9] because inter-individual variation in the number of specific white blood cells in combination with cell specific methylation profiles could compromise measurement outcomes for DNA methylation carried out on cells from whole blood. This concern has largely been theoretical due to lack of experimental data. Recently, Talens *et al.* studied the effect of inter-individual differential white blood cell counts on methylation measurements using whole blood DNA [10]. For a majority of the 16 loci studied, cellular heterogeneity had no effect on variation in DNA methylation. However, for one locus it explained 25–50% of the variation and in three additional loci the effect was borderline significant, accounting for up to 8% of the variation between individuals.

In the present study we aimed to investigate the potential confounding effect of cellular heterogeneity on DNA methylation measurement outcomes conducted using whole blood DNA using the following approach; first measuring methylation levels in whole blood DNA samples and estimating their association with cellular heterogeneity (i.e., proportional white blood cell counts of neutrophils, lymphocytes, monocytes, eosinophils and basophils, counted using an automated cell counter), and subsequently measuring and comparing DNA methylation levels in two whole blood cell fractions, MNCs and PMNCs, in order to verify whether any observed association was related to differential DNA methylation in the white blood cells. These analyses have been done in a general context, rather than in any disease-specific context, in order to understand the overall potential for confounding. A confounding effect may be region-specific, depending on two factors; first, the size of the difference in methylation level between cell types, and second due to the relative size of the difference compared to the variation in methylation levels caused by other factors. We therefore chose to analyze DNA methylation in four CGIs, (or more specifically, parts of CGIs, spanning 4–10 CpG sites in each), which represented a range of inter-individual variation in DNA methylation from very low to very high (in genes *HHEX* (Ensembl identifier: ENSG00000152804), *KCNJ11* (ENSG00000187486), *KCNQ1* (ENSG00000053918) and *PM20D1* (ENSG00000162877)). Our analysis detects region-specific differential DNA methylation in white blood cell fractions and suggests that such difference can confound DNA methylation measurement outcomes conducted on whole blood.

## Results

### DNA methylation in whole blood

**Figure 1:**
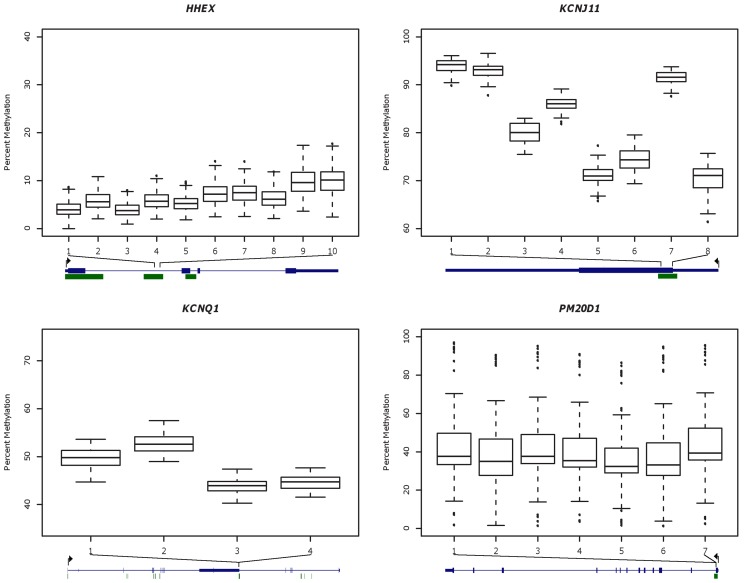
Percent DNA methylation in whole blood samples. Percent DNA methylation (y-axis) in whole blood DNA per CpG site (x-axis) in four CGIs located in the *HHEX* (n = 169), *KCNJ11* (n = 54), *KCNQ1* (n = 49) and *PM20D1* (n = 59) genes respectively. Data for each CGI are depicted in a separate boxplot. Below each boxplot is a gene-map which roughly indicates the position of the analyzed CpG sites (adapted from the UCSC genome browser) [12]. Genes are depicted in blue, the exons as blocks, the introns as thin lines connecting the blocks, and the 5′ and 3′ untranslated regions as thin blocks at each end. CGIs are shown as green blocks. The genomic position depicted for each CGI is; 10:94,439,661–94,445,388 (chromosome:first base-last base) for the *HHEX* CGI, chr11:17,363,372–17,366,783 for the *KCNJ11* CGI, chr11:2,422,797–2,826,916 for the *KCNQ1* CGI and chr1:204,063,776–204,085,881 for the *PM20D1* CGI. The gene map for *KCNQ1* includes the *KCNQ1OT1* (*KCNQ1* overlapping transcript 1) gene, which appears as a large exon roughly in the middle of the map. Arrows indicate the direction of transcription and the position of the transcription start site.

DNA methylation was measured in DNA isolated from whole blood, in CGIs located in four genes; *HHEX*, *KCNJ11*, *KCNQ1* and *PM20D1*. The examined loci are referred to as the “*HHEX* CGI”, ” *KCNJ11* CGI”, “*KCNQ1* CGI” and the “*PM20D1* CGI” in the text below, as the analysis is focused on the methylation levels of the CpG islands, rather than on the genes themselves. We analyzed the methylation levels in a total of 10 CpG sites for the *HHEX* CGI, 8 for the *KCNJ11* CGI, 4 for the *KCNQ1* CGI and 7 for the *PM20D1* CGI. The *HHEX*, *KCNJ11* and *KCNQ1* CGIs had been studied previously at the Icelandic Heart Association (unpublished data) and were chosen to represent low to intermediate variability regions while the *PM20D1* CGI was selected from our previous, published work to represent a highly variable region [11]. More specifically, the CGIs were selected from a larger set of CGIs based on two criteria. First, on basis of the size of the inter-individual variability present at each CGI so as to select CGIs representing a spectrum of variability from very low to very high and second, on basis of which CGI in each variability category had available data on DNA methylation in the largest number of whole blood DNA in our database. The whole blood DNA methylation data used for the present study were thus partly obtained from previous, unpublished studies conducted at the Icelandic Heart Association. Data from all individuals that met the inclusion criteria for the present study were used. The number of samples assayed per CGI was therefore dependent on the sample numbers used in these previous studies that met the inclusion criteria, and thus the sample numbers are unequal and sample overlap between CGIs is incomplete (Figure S1). In total, whole blood DNA methylation data were successfully obtained for 169 individuals for the *HHEX* CGI, 54 for the *KCNJ11* CGI, 49 for the *KCNQ1* CGI and 59 for the *PM20D1* CGI after exclusion of individuals due to missing values and outliers (total sample numbers prior to exclusion were 179, 64, 50 and 59 respectively, see Figure S1 and Table 1). The average age of the 211 individuals included in the study was 75±12 years and 45% were males (Table 1). The average age of the individuals assayed for whole blood DNA methylation in the *HHEX* CGI was about 10 years higher than the average age of the individuals assayed for the other three CGIs (Table 1). Each CpG site was numbered sequentially on the basis of its distance from the forward primer. The exact genomic position and corresponding number assigned to each site is listed in Table S1 and a gene-map for each locus, to indicate the approximate position of the CpG sites analyzed is shown in Figure 1.

**Table 1:** Characteristics of individuals included in the study*.

				Proportional white blood cell count				
	n	Age (years)	%Males	%LY	%MO	%NE	%EO	%BA
Total WB	211	75±12	45	29.7±7.7	9.4±2.9	56.8±8.3	3.6±2.2	0.5±0.5
*HHEX* (WB)	179	75±13	46	28.9±7.3	9.5±3.1	57.6±8.2	3.6±2.2	0.5±0.5
*KCNJ11* (WB)	64	66±17	45	31.5±8.1	9.2±2.4	55.6±8.4	3.3±1.9	0.4±0.7
*KCNQ1* (WB)	50	65±19	52	30.1±7.1	9.4±2.4	56.7±8.0	3.4±1.7	0.4±0.7
*PM20D1* (WB)	59	66±18	53	30.5±7.5	9.6±2.5	55.9±8.1	3.5±1.7	0.4±0.7
Total BCF	20	45±13	50	32.5±5.6	9.1±2.3	54.7±6.3	3.2±1.4	0.4±1.1

* Abbreviations; WB:Whole blood (i.e., population studied for DNA methylation in whole blood), BCF: Blood cell fraction (i.e., population studied for DNA methylation in blood cell fractions), NE: Neutrophils, LY: Lymphocytes, MO: Monocytes, EO: Eosinophils, BA: Basophils.

Whole blood DNA methylation levels differed between CpG sites within each CGI (Figure 1), but the levels were generally very low for the *HHEX* CGI (<20%), intermediate for the *KCNQ1* CGI (ranging between ∼40–60%), intermediate to very high for the *KCNJ11* CGI (ranging between ∼60–100%) and very low to very high for the *PM20D1* CGI (ranging between ∼0–100%). The results also indicated that intra-individual variability in DNA methylation across the respective CpG sites differed between CGIs; it was higher for the *KCNJ11 and KCNQ1* CGIs than for the *HHEX and PM20D1* CGIs. In general, inter-individual variability was lowest for the *KCNQ1* CGI and highest for the *PM20D1* CGI; the standard deviation per CpG site ranged between 1.4–1.9 percentage points (pp) for the *KCNQ1* CGI (1.7 pp on average across all respective CpG sites), 1.5–3.0 pp for the *HHEX* CGI (2.1 pp on average), 1.3–3.4 pp for the *KCNJ11* CGI (2.0 pp on average) and 22.8–25.3 pp for the *PM20D1* CGI (24.3 pp on average).

The inter-individual variability in whole blood DNA methylation level could in theory, at least partly, be explained in terms of differential white blood cell composition between the studied individuals. The numbers of white blood cells, neutrophils, lymphocytes, monocytes, eosinophils and basophils, were counted using an automated cell counter in blood samples drawn from the 211 individuals included in the study (these samples were collected separately at the same time as the blood used for DNA isolation). The white blood cell counts varied considerably between individuals. The average proportion ± standard deviation of the five cell sub-types, in decreasing order, was 56.8%±8.3pp for the neutrophil proportion (the relative standard deviation (RSD): 14.7%), 29.7%±7.7pp for the lymphocyte proportion (RSD: 25.8%), 9.4%±2.9pp for the monocyte proportion (RSD: 30.8%), 3.6%±2.2pp for the eosinophil proportion (RSD: 61.1%) and 0.5%±0.5pp for the basophil proportion (RSD: 96.9%). These proportions were similar in the four sample populations assayed per CGI (Table 1). We analyzed whether the variation in proportional numbers of specific white blood types were associated with variation in measured DNA methylation levels. Due to the number of statistical tests performed for this analysis, a stringent α value of 0.001 was used. Statistical analysis, adjusting for age and gender, indicated that a significant proportion of the variability in the *HHEX* CGI could be explained by heterogeneity in proportional white blood cell counts, or up to 40% (p<0.0001, Table 2). None of the five white blood cell ratios were significantly associated with measurement outcomes for the *KCNJ11*, *KCNQ1* and *PM20D1* CGIs (Table 2). These results were minimally affected by outliers and missing values. We tested for confounding of the results due to batch effects (see materials and methods for details), but none were detected. Finally, an analysis was performed, testing for association between DNA methylation and cellular heterogeneity as before, but using only data from the 29 individuals that were analyzed in all CGIs (Figure S1), to test whether the use of different sample subsets affected the results. Again, no association was observed between DNA methylation in the *KCNJ11*, *KCNQ1* and *PM20D1* CGIs and cellular heterogeneity and DNA methylation in the *HHEX* CGI was associated with lymphocyte and neutrophil proportions (only), and the effect sizes were similar to the previous analysis using all available data.

**Table 2:** Proportion of variation in measured whole blood DNA methylation level accounted for by cellular heterogeneity.

	Variance explained by cell proportion (%)				
CGI	Lymphocytes	Monocytes	Neutrophils	Eosinophils	Basophils
*HHEX*	40*	0	29*	0	0
*KCNJ11*	0	0	0	0	3
*KCNQ1*	3	0	1	0	0
*PM20D1*	0	0	0	0	0

* p<0.001.

### DNA methylation in white blood cell fractions

To examine if the variability in measured methylation level at different CGIs in whole blood was attributable to differential methylation in the white blood cell types comprising whole blood, we fractionated whole blood samples from 20 individuals into mononuclear cells (MNCs, containing lymphocytes and monocytes) and polymorphonuclear cells (PMNCs, containing neutrophils, basophils and eosinophils), isolated DNA and measured the methylation levels at the four CGIs in each fraction. The average age of the 20 individuals was 45±13 years and 50% were males (Table 1). DNA was also isolated from whole blood for these individuals, methylation levels measured in each of the four CGIs and the data included in the analysis above.

**Figure 2:**
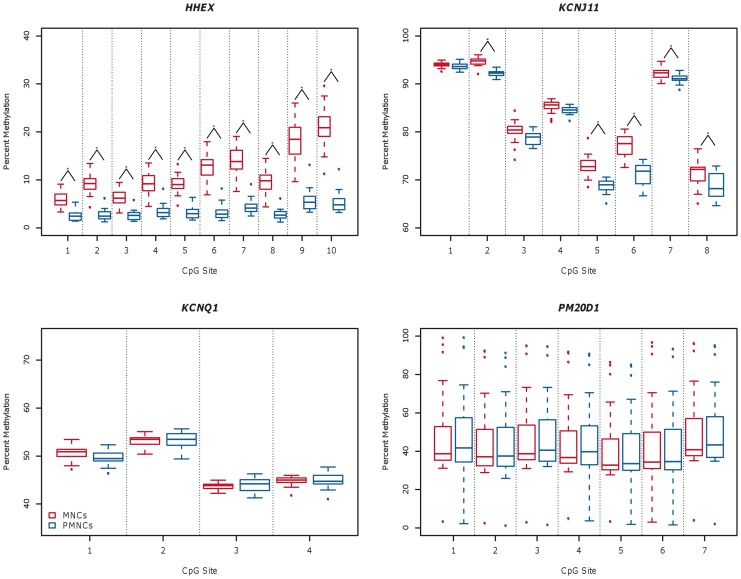
Percent DNA methylation in mononuclear and polymorphonuclear cells. Percent DNA methylation (y-axis) in mononuclear and polymorphonuclear cells (MNCs and PMNCs) per CpG site (x-axis) in four CGIs located in the *HHEX*, *KCNJ11*, *KCNQ1* and *PM20D1* genes respectively (n = 20 each). Data for each CGI are depicted in a separate boxplot where measurements for MNCs are shown in red and for PMNCs in blue. The dotted lines separating the boxes indicate that at each CpG site a pair of data are being compared (i.e., for MNCs and PMNCs). Significantly (p<0.01) differentially methylated CpG sites (MNCs versus PMNCs DNA methylation) are indicated with an asterisk.

We compared the methylation levels measured in MNCs and PMNCs and observed a higher average methylation in MNCs in 21 of the 29 CpG sites analyzed in total. Paired Wilcoxon signed rank test revealed that 15 of these CpGs were significantly (α = 0.01 was used due to number of statistical tests performed) differentially methylated in the two different cell fractions, located in the *HHEX* and *KCNJ11* CGIs (Figure 2). The average absolute difference between the two cell fractions was highest for the *HHEX* CGI. All ten CpGs studied at this CGI showed significantly higher methylation in MNCs. The absolute difference ranged between 3.4–15.7 pp (corresponding to ∼2.3–4.0 fold higher methylation levels in MNCs per CpG site). DNA methylation in the *KCNJ11* CGI was also generally higher in MNCs. The difference was more moderate, but nonetheless significant in 5 out of 8 CpGs, ranging between 0.4–6.1 pp (corresponding up to ∼1.1 fold higher methylation levels). DNA methylation levels did not differ significantly in the *KCNQ1 and PM20D1* CGIs. Comparable results were obtained by comparing average DNA methylation across all respective CpG sites per CGI between the two cell groups; the average DNA methylation levels differed significantly in the *HHEX* and *KCNJ11* CGIs, but not in the *KCNQ1* and *PM20D1* CGIs. In the *HHEX* CGI, average methylation levels across the 10 CpG sites were higher by 8.0 pp in MNCs (p = 8·10^−6^) and by 2.4 pp in the *KCNJ11* CGI (p = 2·10^−5^).

### DNA methylation levels are correlated between blood cell fractions

**Figure 3:**
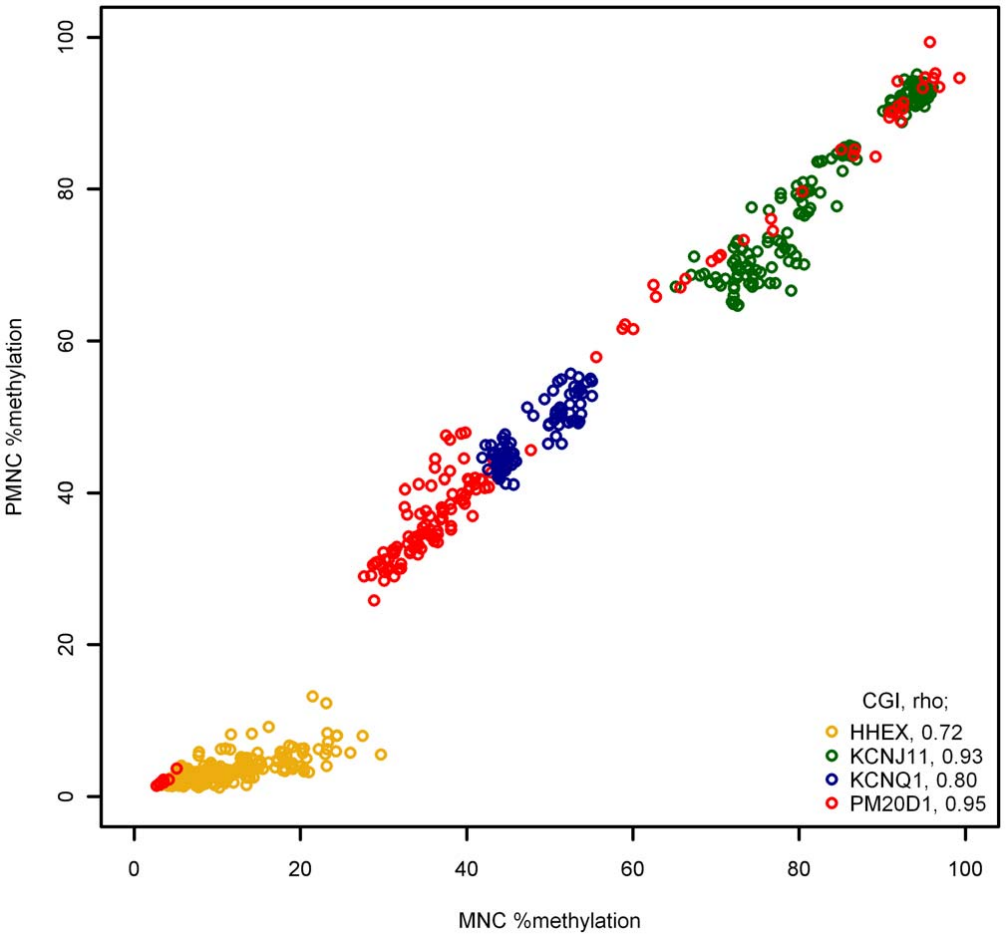
Correlation between DNA methylation in mononuclear and polymorphonuclear cells. Comparison of DNA methylation levels measured in two cell fractions, mononuclear cells (MNCs) and polymorphonuclear cells (PMNCs). Percent methylation in PMNCs (y-axis) is plotted against percent methylation in MNCs (x-axis). Each dot represents the two measurements for a single CpG per individual. The Spearman ρ for correlation between measurements in MNCs and PMNCs for each CGI is shown in the legend.

The results in Figure 2 suggest that the methylation patterns between cell fractions are highly similar. To quantify this observation we analyzed the correlation between methylation levels for the two different fractions (Figure 3). The correlation was very high in all CGIs, irrespective of whether methylation levels differed between cell fractions or not. Spearman's ρ was 0.72 for *the HHEX* CGI, 0.93 for the *KCNJ11* CGI, 0.80 for the *KCNQ1* CGI and 0.95 for the *PM20D1* CGI.

## Discussion

Studies on DNA methylation using whole blood DNA frequently do not control for inter-individual variation in the cellular population from which the DNA is derived, the white blood cells; lymphocytes, neutrophils, eosinophils, basophils and monocytes. This has been criticized due to hypothesized potential for confounding effect when cellular heterogeneity is present in conjunction with cell type specific DNA methylation [9]. Here, we studied this hypothesis by first testing for an association between whole blood DNA methylation levels and cellular heterogeneity, and second to test whether differential methylation in two cell fractions might underlie the observed association. Our data indicated that indeed a locus specific association between measured DNA methylation levels and cellular heterogeneity in whole blood can be observed. Further, we observed significant differences in locus specific DNA methylation levels in two blood fractions, MNCs and PMNCs, suggesting that it could be the underlying cause of the observed association between DNA methylation levels and white blood cell counts. Finally, in all loci tested we observed that DNA methylation in MNCs and PMNCs is highly correlated independent of differential methylation levels in these fractions.

Up to 40% of the inter-individual variation in whole blood DNA methylation in the *HHEX* CGI was attributed to cellular heterogeneity, suggesting that a considerable confounding can affect measured levels of whole blood DNA methylation due to differences in the cellular population. No significant effect on measurements for the *KCNJ11*, *KCNQ1* and *PM20D1* CGIs was observed, suggesting that this type of confounding does not affect DNA methylation outcomes universally throughout the genome, but may be locus-specific. These results are in concordance with a previous study [10] where out of a total of 16 loci assayed, only a single locus was affected in similar magnitude as the *HHEX* CGI. Together, these studies indicate that while measured DNA methylation levels in some loci may be affected by cellular heterogeneity, a substantial proportion of loci may not be affected by this confounding effect.

We detected that DNA methylation levels in PMNCs and MNCs differed significantly in two out of four CGIs examined; i.e., in all CpG sites analyzed in the *HHEX* CGI and 5 of 8 CpG sites analyzed in the *KCNJ11* CGI but not in the *KCNQ1* and *PM20D1* CGIs. The gross difference observed in the *HHEX* CGI may reflect the fact that the *HHEX* gene is differentially expressed in the various blood cells [13]–[15]. Just as in whole blood DNA methylation measurements, this analysis may have been confounded by cellular heterogeneity because PMNCs and MNCs both consist of groups of cells. However, the fractionation split up the two white blood cell groups that affected whole blood DNA methylation measurements and their numbers are so dominant relative to the other groups that the analysis is likely to be minimally affected. Kerkel *et al.* have previously studied methylation in these fractions, and identified multiple differentially methylated loci [16]. Their analysis was however not described in detail. Nonetheless, together these studies indicate that differential methylation between white blood cell types may be relatively common. Further supporting this assumption, a study published after the initial submission of our report includes an investigation of the DNA methylation levels in about 27 thousand CpG sites across the genome in multiple types of white blood cells (specifically multiple T cell subtypes, natural killer cells, B cells, neutrophils and monocytes) which identified ∼40% of the CpG sites as significantly differentially methylated among the different cell types [17]. The size of the difference was only described for a relatively small number of the CpG sites analyzed and their potential effect on whole blood DNA methylation measurements at the respective CpG sites therefore cannot be deduced. A second study from this research group (also published after initial submission of our report) demonstrates a method for quantifying the composition of white blood cells from whole blood DNA methylation levels at certain loci, underscoring the relationship between DNA methylation levels in whole blood and proportional white blood cell counts which we have investigated [18].

Analysis of DNA methylation both in whole blood and blood fractions has allowed evaluation of the hypothesis that measured DNA methylation levels in whole blood can be confounded by cellular heterogeneity due to differential methylation levels in the various white blood cell types. We observed differential methylation between cell fractions in the *HHEX* and *KCNJ11* CGIs and not in the *KCNQ1* and *PM20D1* CGIs, but we were only able to detect a significant effect due to cellular heterogeneity on whole blood DNA methylation measurement outcomes for the *HHEX* CGI. However, the difference in DNA methylation between fractions was very moderate in the *KCNJ11* CGI. It is therefore possible that the effect of cellular heterogeneity on measurement outcomes for the *KCNJ11* CGI, if any, is exceedingly subtle, and thus undetectable by the methods we employed. It is therefore our view that these results support the hypothesis, and that they suggest a need to control for cellular heterogeneity in the analysis of methylation in blood cells.

Since the confounding effect would only be observed when both the genomic region of interest is differentially methylated amongst white blood cell types, and when there is blood cell count heterogeneity in the individuals being compared, controlling for this problem may be addressed in different ways depending on available data. Differences in white blood cell composition may be assessed if blood cell counts for the individuals under investigation are available. Alternatively, subjects can be paired with controls that are concordant in terms of cellular composition prior to the analysis. Furthermore, whole blood can be fractionated to assess possible differential methylation in the area of interest. This may be done with the Ficoll medium method used here which is relatively easy to perform, but due to heterogeneity in the fractions, as noted previously, this approach may not be sufficient to address the problem. Finally, referring to the literature may be advisable to assess the risk of altered blood cell counts in the groups of individuals under study. For example white blood cell counts have been shown to be associated with the development of cancers [19] and coronary heart disease [20]. This raises the issue that whenever there is a difference in cell fractions associated with disease, an adjustment for blood cell proportions could be essential for better controlled analyses.

The different approaches may cause inconsistent results, and therefore it is important to standardize methods for this correction. As has been discussed previously [10], adjusting for white blood cell counts can be achieved with standard statistical approaches. Such an approach may be well suited for that purpose since such data are presumably readily available at many laboratories conducting experiments on whole blood DNA. This could be achieved in two ways: One is to use multiple variables accounting for the absolute number of each cell type (commonly five; neutrophils, lymphocytes, monocytes, basophils and eosinophils) or alternatively use a single variable accounting for the proportion of one cell type. Using a single variable is more appealing because the other option would reduce the number of degrees of freedom. However, to be able to correct for the confounding effect of cellular heterogeneity in statistical models by using a variable accounting for the proportional number of one cell type, there needs to be a correlation between methylation levels in the different types of white blood cells. Our results indicate that in the analyzed CGIs, methylation patterns across the corresponding CpG sites within a CGI are very similar between the different cell types irrespective of demonstrable differences in the cell specific absolute methylation levels. Our analysis therefore suggests that use of a single variable to account for the proportional number of a single cell type (e.g., neutrophils or lymphocytes) in statistical analyses might be sufficient to correct for the confounding effect of cellular heterogeneity on DNA methylation measurements conducted using whole blood DNA.

The principal limitation of the present study is the small number of CGIs assayed, which prevents us from making generalized claims of the characteristics (frequency, etc.) of confounding due to cellular heterogeneity. The study does however highlight the potential for such confounding, as was its purpose. A second limitation of the study is that we fractionated whole blood into two cell groups, PMNCs and MNCs. We therefore cannot definitively claim that in the CGIs where little or no differential methylation was detected, that they are not differentially methylated if all the various white blood cell types were compared. Finally, a third limitation is that for the experiment testing for association between DNA methylation levels in whole blood and cellular heterogeneity, sample sizes were unequal, and sample overlap incomplete between CGIs. This does however not appear to jeopardize our results; when only the 29 individuals that had DNA methylation data for all four CGIs were included in this analysis, comparable results were obtained.

Our findings may not only be relevant for methylation measurements using whole blood DNA. Other tissues are samples of different types of cells as well, so a similar problem could affect measurements in these tissues. Our data indicate that although methylation levels may differ between blood cell types in some loci, the methylation pattern may at the same time be very similar (as indicated by the high correlation between methylation levels). This is in agreement with previous studies which have shown that different cells and tissues, even from separate germ layers, generally have similar DNA methylation patterns [2], [10], [21]. If blood cell DNA methylation measurements could be used as surrogates for methylation in other tissues based on this feature, it might be preferable to use blood.

DNA methylation levels are sometimes assessed in a global manner, assaying CpG sites across the entire genome. Since our study was conducted in a gene-specific manner the results may not apply to global DNA methylation measurements. Indeed, in a previous study using LUMA to estimate global methylation, we report no association between methylation levels and white blood cell counts [22]. However, as mentioned above, Wu *et al.* report that global methylation levels in PMNCs, as measured by LUMA, are significantly higher than in MNCs and are not correlated [8]. In the same study, results from three other assays for global DNA methylation showed no association between PMNCs and MNCs methylation levels. It is therefore possible that global methylation measurements are also confounded by cellular heterogeneity. A more detailed analysis, including comparison on the association between global methylation levels in whole blood and cellular composition, such as in the present study, should be conducted in order to extend these observations.

The results from the present study call for an analysis of larger number of CpG sites to reveal the full extent of how confounding effects may influence analyses on DNA methylation conducted using whole blood DNA. It is important to assess whether measured methylation levels at a considerable number of loci are affected by this effect. Second, it would be of value to study whether methylation of CpGs positioned in certain genes is more prone to be affected by this factor than others (e.g., in genes that are differentially expressed in the different cell subtypes such as *HHEX*). Finally, it would be interesting to investigate whether certain sequences (e.g., introns, exons, CGIs, CGI shores, transcription start sites or promoter regions) are more likely to be affected by this confounding effect.

## Materials and Methods

### Ethical statement

The Age, Gene/Environment Susceptibility (AGES)-Reykjavik [23] and the Risk Evaluation For Infarct Estimates (REFINE)-Reykjavik studies are approved by the Icelandic National Bioethics Committee (VSN: 00-063, VSN: 05-112 respectively) and the Data Protection Authority. All participants gave written informed consent on arrival to the clinic.

### Samples

Samples used in the present study were obtained from two cohort studies conducted at the Icelandic Heart Association, the AGES-Reykjavik [23] and the REFINE-Reykjavik studies. Whole blood DNA samples, which were analyzed independently for each CGI assayed in the study, were obtained from both the AGES-Reykjavik and the REFINE-Reykjavik studies (n = 191). Blood was collected from individuals taking part in the REFINE-Reykjavik study (n = 20), and these samples subsequently used for DNA extraction from both whole blood and two whole blood cell fractions (see details in next section). Three DNA samples were therefore obtained from each blood sample. All three DNA samples from all the 20 individuals were analyzed for each of the four CGIs assayed in the study. Both the whole blood DNA samples we obtained and the blood samples we collected were randomly selected from apparently healthy men and women. The age range of all individuals included in the study (n = 211) was 22–96 years, and ∼45% were males (further details are provided in Table 1). An overview of the total number of whole blood DNA samples analyzed per CGI, and their overlap is provided in Figure S1.

Briefly, AGES-Reykjavik study was the seventh visit in the Reykjavik Study, a population-based cohort study initiated in 1967, inviting all Reykjavik inhabitants born between 1907 and 1935 to participate. In this visit, 5764 of the surviving members were recruited. REFINE-Reykjavik is a prospective study on risk factors and cause of atherosclerotic disease in a population of Icelandic people. The main goal of the study is to improve the predictability of cardiovascular disease risk estimates. The study was initiated in 2005 and recruitment of the first phase was completed in spring 2011 recruiting 6942 men and women born in the years 1936–1980 living in the Reykjavik city area.

### DNA isolation

Whole blood was fractionated by density gradient centrifugation using Histopaque-1077 Ficoll medium and Accuspin™ Tubes (Sigma-Aldrich, catalog numbers (cat.nr.): 10771 and A1930 respectively). The mononuclear cell fraction was extracted from the serum/medium boundary and the polymorphonuclear cell fraction from the bottom of the tubes. The blood samples were processed as “fresh” as possible, never later than 4 hours after the blood draw.

A simple salting out method was used for DNA extraction, based on an extraction method developed by Scotlab Bioscience (Coatbridge, Scotland, UK). The DNA was dissolved in TE buffer and its concentration measured using UV absorbance quantification (260 nm) on a Spectramax M2 (Molecular Devices, Sunnyvale, CA, USA) microplate reader.

### Blood cell counts

For all participants, white blood cells (monocytes, lymphocytes, eosinophils, basophils and neutrophils) were counted in whole blood by an automated cell counter, Coulter HmX AL Hematology Analyzer (Beckman Coulter, High Wycombe, England, UK).

### Bisulfite conversion of DNA samples

Bisulfite conversion of DNA samples was carried out using the EZ DNA Methylation™ kit (Zymo Research, cat.nr.: D5004) following the manufacturer's instructions. When the DNA was not analyzed immediately following the conversion process it was stored at −20°C for later use. DNA from blood fractions and the corresponding whole blood DNA for each individual was converted in the same batch.

### Analysis of DNA methylation

Assays were designed to analyze DNA methylation levels in CGIs, located using the University of California, Santa Cruz genome browser (Human March 2006 NCBI36/hg18 assembly) [12]. Primer sets (forward and reverse PCR primers, one tagged with biotin, and a sequencing primer) were designed using PyroMark Assay Design software (version 2.0.1.15, QIAGEN, Hilden, Germany). Primer sequences and genomic positions of the CpG sites analyzed in each assay are listed in Tables S1 and S2. A 30 µl PCR was carried out on a 2720 Thermal cycler (Applied Biosystems, Foster City, CA, USA) using 1× TITANIUM Taq polymerase (Clontech, cat.nr.: 639220) or 3 units OneTaq™ Hot Start polymerase (New England Biolabs, cat.nr.:M0481L), 1× Standard Taq Reaction Buffer (New England Biolabs, cat.nr.: B90145), 0.2 mM dNTP (New England Biolabs, cat.nr.: N04465), 0.25 µM of each primer (Sigma-Aldrich) and 3 µl of bisulfite converted DNA. PCR cycling conditions for all assays were as follows; 2 minutes at 96°C, followed by 40 cycles of 90 s at 96°C, 90 s at 62°C and 90 s at 72°C and finally 72°C for 10 minutes after cycling.

The biotinilated sequencing template was extracted from the PCR product mixture by annealing with streptavidin coated sepharose beads (Streptavidin Sepharose™ High Performance, GE Healthcare, cat.nr.: 17-5113-01). The template was subsequently washed in a series of steps using a Vacuum prep workstation (QIAGEN cat.nr.: 9001518) and finally released onto a sequencing plate (QIAGEN, cat.nr.: 979201) containing annealing buffer (QIAGEN, cat.nr.: 979309) with the appropriate sequencing primer. The samples were analyzed for methylation at each CpG site using a PyroMark Q24 pyrosequencer (QIAGEN) and PyroMark™ Gold Q24 reagents (QIAGEN, cat.nr.: 97082).

### Data analysis

Pyrograms from the pyrosequencing reactions were analyzed with the “PyroMark Q24 Software” (v1.0.10, QIAGEN). Methylation levels were calculated as the ratio between peak heights for methylated C's and the sum of methylated and unmethylated C's for each CpG site. Default software settings were used for quality assessment of the pyrograms per CpG site and measurements that failed the assessment were discarded when appropriate. Consequently, some individuals had missing values for one or more CpG site and were analyzed separately. The cause of failed quality assessment was dominantly low signal strength. To verify that the assays were robust, the measurements were partly replicated (analysis not shown). For the replicated data, average methylation from the two measurements was used in the subsequent analysis. We tested for batch effects introduced by use of two brands of polymerases and only pooled data acquired through use of the two polymerases lacking any significant batch effects (data not shown). Finally, outliers were defined as values outside mean±2.698 s (where s is standard deviation) per CpG site. For a standard Gaussian distribution, this criterion defines 0.35% of the data farthest from the mean in both directions as outliers. Individuals with one or more outliers were analyzed separately to prevent potential sporadic measurement error affecting the analyses. For all statistical analyses, uncorrected p-values were reported.

In total, whole blood DNA methylation data were obtained for 179 individuals for the *HHEX* CGI, 64 individuals for the *KCNJ11* CGI, 50 individuals for the *KCNQ1* CGI and 59 individuals for the *PM20D1* CGI (Figure S1). For the *HHEX* CGI, one or more outliers were detected in the CpGs studied for seven individuals, and measurement of methylation at one or more CpG sites failed the quality assessment in an additional three samples. For the *KCNJ11* CGI, measurements for five samples failed quality assessment at one or more CpG sites and five outliers were present. For the *KCNQ1* CGI, a single outlier was present, but no missing values. No outliers were present in the data for the *PM20D1* CGI and none of the measurements failed the quality assessment. Successful and reliable measurements for all corresponding CpG sites in 169 samples for the *HHEX* CGI, 54 for the *KCNJ11* CGI, 49 for the *KCNQ1* CGI and 59 for the *PM20D1* CGI were therefore obtained from whole blood DNA and used in the subsequent analysis. The proportion of variation in methylation levels between individuals explained by differential white blood cell counts was estimated from mixed model analysis of the data using PROC MIXED in SAS Enterprise Guide version 4.2 using a random intercept term to account for the correlation within a person. To approach homoscedasticity, the whole blood DNA methylation data were transformed by taking the arcsine of the square root of the percentages. Since R^2^ cannot be obtained directly from random effects models, two models were applied, a base model containing CpG sites and covariates (age and sex) as fixed effects and a full model where additionally, the proportional number of a specific cell type was added to the base model as fixed effect. R^2^ was then calculated from the residual variance (v_ri_ and v_rf_ for the base and full models respectively) and variance of the random intercept (v_si_ and v_sf_ for the base and full models respectively) terms using the formula R^2^ = (V_i_−V_f_)/V_i_ where V_i_ = v_ri_+v_si_ for the base model and V_f_ = v_rf_+v_sf_ for the full model. To test for confounding of our results due to batch effects, covariates for sample populations, conversion batch and date of analysis on the pyrosequencer were added to the models. Due to the number of statistical tests performed for this analysis, testing for association between DNA methylation in whole blood and proportional white blood cell counts, a stringent α value is appropriate. Assuming that DNA methylation levels in the four CGIs investigated are independent and that the five cell proportions are correlated necessitates a correction by a factor of four. The analysis was repeated four times, adjusting for different cofactors and using various subpopulations of the samples. Therefore α = 0.001 (α = 0.05/4/4≈0.001) was considered appropriate for this particular analysis.

Measurements on DNA from the two blood cell fractions, PMNC and MNCs, were conducted on the same 20 individuals (40 samples total) for all four CGIs. Measurements were successfully obtained for all CpG sites at all CGIs. A single outlier was present in the data for the *HHEX*, *KCNJ11* and *KCNQ1* CGIs, but none in the data for the *PM20D1* CGI. Due to the limited number of samples used in this analysis, these data were not excluded. Excluding the data did however not affect the analysis (analysis not shown). The data were analyzed using non-parametric statistics to avoid making a generalized assumption about the distribution of our data, which may differ between loci. Paired Wilcoxon signed rank test was used to assess statistical differences in methylation levels between the two cell populations and their correlation assessed with Spearman correlation coefficient using R version 2.12.2. Due to the number of statistical tests performed for the analysis comparing differences in methylation levels between the two cell populations a more stringent α value than 0.05 was considered appropriate. The apparently high correlation of DNA methylation levels between CpG sites within CGIs (Figure 3) argues against correcting for each comparison of individual CpGs within a CGI, but a correction for the number of CGIs investigated (four) is necessary. Therefore α = 0.01 (α = 0.05/4≈0.01) was considered appropriate for this particular analysis.

## Supporting Information

Figure S1 **Venn diagram depicting the number of samples analyzed per CGI.** The diagram contains a set of 15 numbers that, when added together, represent the total number of individuals analyzed with DNA from whole blood. Each ellipse contains a set of numbers, that when added together represent the total number of individuals analyzed for a specific CGI. Finally, some individuals were analyzed for more than one CGI, and this is represented by the overlapping of ellipses.
(FIFF)

Table S1 **Genomic positions of the CpG sites analysed per locus.**
(DOC)

Table S2 **List of primer sequences used for the PCR amplification.**
(DOC)
