# Supplementary figures and images for: Heterogeneity in White Blood Cells Has Potential to Confound DNA Methylation Measurements
Source: PLoS One. 2012 Oct 5;7(10):e46705. doi: 10.1371/journal.pone.0046705 (PMC3465258; doi:10.1371/journal.pone.0046705)

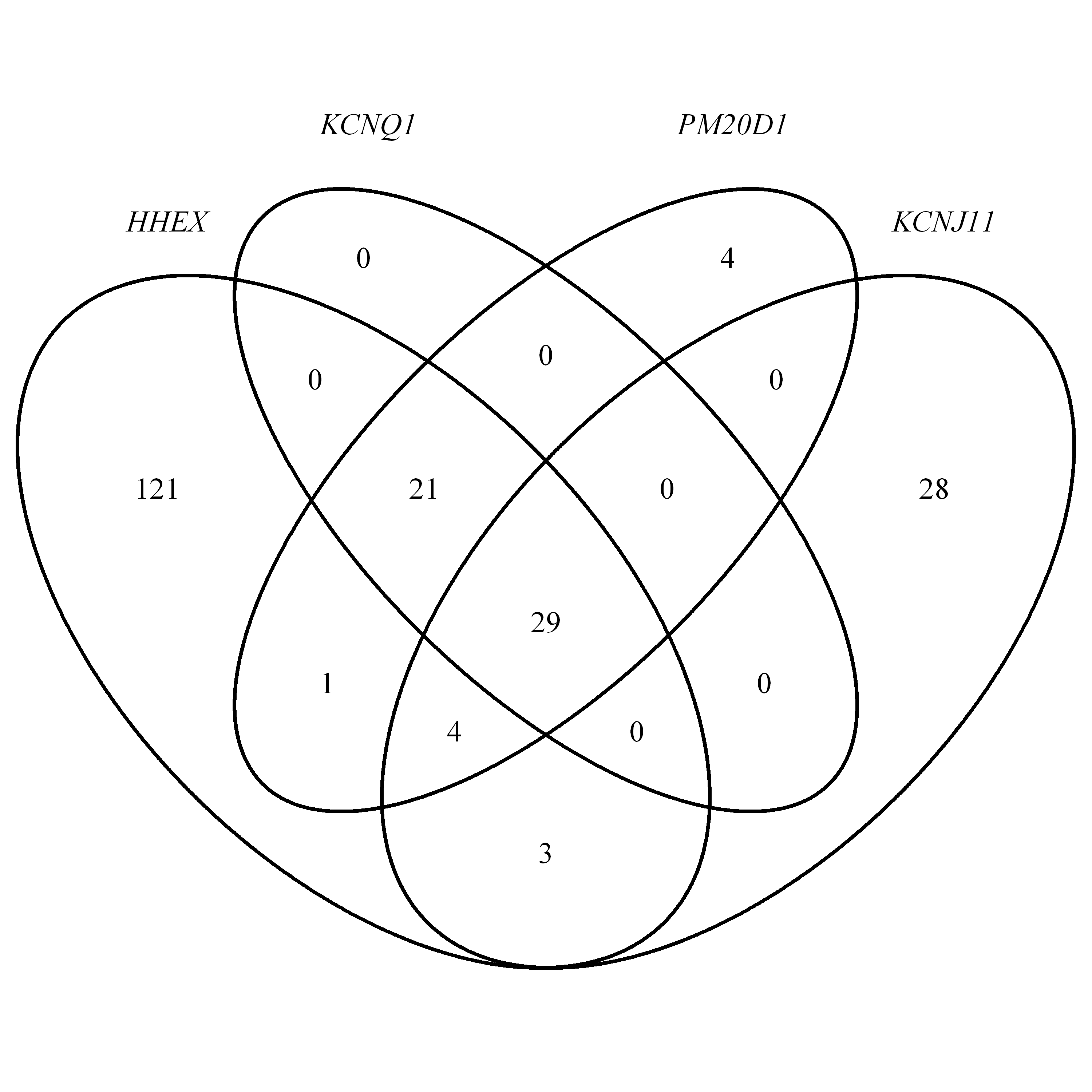

Supplement: Figure S1 — Venn diagram depicting the number of samples analyzed per CGI. The diagram contains a set of 15 numbers that, when added together, represent the total number of individuals analyzed with DNA from whole blood. Each ellipse contains a set of numbers, that when added together represent the total number of individuals analyzed for a specific CGI. Finally, some individuals were analyzed for more than one CGI, and this is represented by the overlapping of ellipses. (FIFF) [file pone.0046705.s001.fiff]
