# Supplementary material for: Heterogeneity in White Blood Cells Has Potential to Confound DNA Methylation Measurements
Source: PLoS One. 2012 Oct 5;7(10):e46705. doi: 10.1371/journal.pone.0046705 (PMC3465258; doi:10.1371/journal.pone.0046705)
Supplement: Table S1 — Genomic positions of the CpG sites analysed per locus. (DOC) [file pone.0046705.s002.doc]

**Table S1.** **Genomic positions* of the CpG sites analysed per locus.**

|  |  | **Position** | | | | | | | | | |
| --- | --- | --- | --- | --- | --- | --- | --- | --- | --- | --- | --- |
| **CGI** | **Chromosome** | **1** | **2** | **3** | **4** | **5** | **6** | **7** | **8** | **9** | **10** |
| HHEX | 10 | 94441605 | 94441607 | 94441619 | 94441627 | 94441633 | 94441637 | 94441644 | 94441646 | 94441662 | 94441676 |
| KCNJ11 | 11 | 17366204 | 17366187 | 17366178 | 17366168 | 17366135 | 17366129 | 17366123 | 17366114 |  |  |
| KCNQ1 | 11 | 2677095 | 2677111 | 2677115 | 2677117 |  |  |  |  |  |  |
| PM20D1 | 1 | 204085711 | 204085713 | 204085716 | 204085733 | 204085740 | 204085749 | 204085760 |  |  |  |

* Human March 2006 NCBI36/hg18 assembly
