# Supplementary material for: Heterogeneity in White Blood Cells Has Potential to Confound DNA Methylation Measurements
Source: PLoS One. 2012 Oct 5;7(10):e46705. doi: 10.1371/journal.pone.0046705 (PMC3465258; doi:10.1371/journal.pone.0046705)
Supplement: Table S2 — List of primer sequences used for the PCR amplification. (DOC) [file pone.0046705.s003.doc]

**Table S2.** **List of primer sequences used for PCR amplification.**

| **CGI** | **Sequencing primer** | **Forward primer** | **Reverse primer** |
| --- | --- | --- | --- |
| HHEX | GTTAGGATTGGAGGTTT | ATGTTGTTATAGTTTATGGGGTGGT | TTACCCCCTTAAATCTCCCTTAATA |
| KCNJ11 | ATCACCCAAACCATACTATCC | GTTGTAGTTGTTTTTTTTGGATATAAAG | ACTCTACAATAAAACCCTAAACCAC |
| KCNQ1 | GGTTAGGTTGTATTGTTG | GTATTGTTTAGGTTAGGTTGTATTGT | ACCCTCCCCATCTCTCTAA |
| PM20D1 | GTTGAATTGAGAAGGGAT | ATGAGTATAGGTGGGTGAAG | ACCCTAATAACTATACTACTCCTAATTTTC |
